# Cue Valence Influences the Effects of Cue Uncertainty on ERP Responses to Emotional Events

**DOI:** 10.3389/fnhum.2020.00140

**Published:** 2020-04-15

**Authors:** Huiyan Lin, Jiafeng Liang, Ting Liu, Ziping Liang, Hua Jin

**Affiliations:** ^1^Institute of Applied Psychology, School of Public Administration, Guangdong University of Finance, Guangzhou, China; ^2^Laboratory for Behavioral and Regional Finance, Guangdong University of Finance, Guangzhou, China; ^3^School of Education, Guangdong University of Education, Guangzhou, China; ^4^Key Research Base of Humanities and Social Sciences of the Ministry of Education, Center of Cooperative Innovation for Assessment and Promotion of National Mental Health, Academy of Psychology and Behavior, Tianjin Normal University, Tianjin, China

**Keywords:** cue valence, cue uncertainty, positive, negative, ERPs

## Abstract

Individuals often predict consequences, particularly emotional consequences, according to emotional or non-emotional signals conveyed by environmental cues (i.e., emotional and non-emotional cues, respectively). Some of these cues signify the consequences with certainty (i.e., certain cues), whereas others do not (i.e., uncertain cues). Several event-related potential (ERP) studies regarding non-emotional cues have suggested that the effects of cue uncertainty on attention to emotional events occur in both perception and evaluation processes. However, due to the limitations of previous studies, it is unclear what the effects of cue uncertainty would be in an emotional cue condition. Moreover, it is uncertain whether the effects of cue uncertainty are affected by cue valence (i.e., emotional and non-emotional cues). To address these questions, we asked participants to view cues and then to view emotional (positive or negative) pictures. The cues either did or did not indicate the emotional content of the picture. In the emotional cue condition, happy and fearful faces were used as certain cues indicating upcoming positive and negative pictures, respectively, and neutral faces were used as uncertain cues. In the non-emotional cue condition, scrambled faces outlined in red and blue indicated upcoming positive and negative pictures, respectively, and scrambled faces outlined in green served as uncertain cues. The results showed that for negative pictures, ERP responses in a time range between 60 and 1,000 ms were shifted to a more negative direction in a certain condition than in the uncertain condition when the cues were emotional. However, the effect was the reverse for positive pictures. This effect of cue uncertainty was similar in the non-emotional cue—negative condition. In contrast, there was no effect of cue uncertainty in the non-emotional cue—positive condition. Therefore, the findings indicate that cue uncertainty modulates attention toward emotional events when the events are signified by emotional cues. The findings may also suggest that cue valence modulates the effects of cue uncertainty on attention to emotional events.

## Introduction

In everyday life, individuals attempt to predict the consequences of events so that they can approach desirable events and avoid undesirable ones. Their expectations are often based on either emotional or non-emotional signals conveyed by environmental cues. For instance, if you are hiking with a friend in a dark forest, you will expect imminent danger if you see a fearful expression (i.e., an emotional cue) on your friend’s face. The same expectation will occur if you see your friend raising his/her hands, a prearranged signal of danger (i.e., a non-emotional cue). Some of these emotional and non-emotional cues signify upcoming events with certainty (i.e., they are certain cues), whereas others do not (i.e., they are uncertain cues). The different categories of cues might critically influence the perception of emotional consequences and even guide decision-making associated with those consequences (e.g., Onoda et al., 2006, 2007, 2008; Sarinopoulos et al., 2010; Grupe and Nitschke, 2011; Gole et al., 2012; Lin et al., 2012, 2014a,b, 2015a,d, 2017b, 2018; Yang et al., 2012; Aue et al., 2013; Dieterich et al., 2016, 2017; Sussman et al., 2017; Qiao et al., 2018).

Laboratory studies often use a cue-event paradigm to manipulate different categories of cues (e.g., Onoda et al., 2006, 2007, 2008; Sarinopoulos et al., 2010; Grupe and Nitschke, 2011; Staudinger et al., 2011; Gole et al., 2012; Lin et al., 2012, 2014a,b, 2015a,d, 2017b, 2018; Yang et al., 2012; Aue et al., 2013; Gruber et al., 2013; Galli et al., 2014; Dieterich et al., 2016, 2017; Qiao et al., 2018). In this paradigm, expectancy cues are presented before emotional events. Cue uncertainty is manipulated by whether the cues signify the emotional consequence of an event (i.e., certain cues) or do not (i.e., uncertain cues). For emotional cues, the consequence is indicated by the emotional features of the cues (e.g., the negative consequence is indicated by a preceding negative face). Regarding non-emotional cues, the uncertainty of the emotional consequence is indicated by non-emotional features of the cues (e.g., negative consequences are indicated by a preceding leftward arrow, and emotionally uncertain consequences are indicated by a bidirectional arrow).

Theories regarding expectancy bias have suggested that cues indicating an emotional event, particularly a negative event, alter emotional states and thus attention towards the event (Tomarken et al., 1989; de Jong et al., 1995; Davey and Dixon, 1996; Lin et al., 2012; Lin et al., 2014b; Sussman et al., 2017). Consistent with these theories, a behavioral study by Aue et al. (2013) reported slower response times in identifying neutral stimuli when participants had expected the occurrences of negative stimuli than those of neutral stimuli. Sussman et al.’s (2017) functional magnetic resonance imaging (fMRI) study showed that cues indicating upcoming threat increased amygdala activity (a brain region associated with emotional attention) for subsequently presented threat faces. Moreover, studies on uncertainty have shown that attention towards the event, particularly the emotional event, is altered by cue uncertainty due to worry and anxiety, motivation and biased expectancies of negative consequences (e.g., Grupe and Nitschke, 2011, 2013; Dieterich et al., 2016, 2017; for a review, Anselme, 2010). In line with those studies, Sarinopoulos et al. (2010) showed stronger amygdala responses to uncertainty compared to certain negative events. For negative events, the effects of cue (un)certainty might be associated with task demands. For example, when individuals are asked to estimate the probability of the occurrence of a specific emotional category before each event (i.e., explicit expectation), the attention-associated effect regarding the negative event is associated with uncertain cues. When there is no estimation before the event (i.e., implicit expectation), the extents in altered attention are larger for certain cues than for uncertain cues (Lin et al., 2018). However, due to low temporal resolution, behavioral and fMRI measures could not detect in which time ranges the changes of attention occur.

With high temporal resolution, previous studies have tried to use event-related potentials (ERPs) to investigate the time ranges in which attention is influenced by cue uncertainty. ERPs, occurring before 300 ms following stimulus onset, are suggested to reflect attention in the perceptual process (e.g., Luck et al., 1990; Thorpe et al., 1996; Schupp et al., 2003, 2004, 2006; Correa et al., 2006; Talsma et al., 2007; Yuan et al., 2007; Folstein and Van Petten, 2008; Olofsson et al., 2008; Gable and Harmon-Jones, 2012). In later time ranges (>300 ms), the responses of these ERPs were found to reflect attention during emotional evaluations (e.g., Schupp et al., 2000; Hajcak et al., 2006; Moser et al., 2006; Olofsson et al., 2008).

Using the abovementioned ERPs, several studies have shown the effects of cue uncertainty on negative events when the emotional consequence is indicated by non-emotional cues. Note that there are studies regarding both explicit expectations (Dieterich et al., 2016, 2017; Lin et al., 2018; Qiao et al., 2018) and implicit expectations (Gole et al., 2012; Yang et al., 2012; Lin et al., 2015a, 2017b, 2018). Because the present study investigates the effect of cue uncertainty only in the context of implicit expectations, we only introduce the following related studies. In this field of research, Gole et al. (2012) found that ERP responses were more positive-going for negative pictures after certain cues (i.e., in the certain condition) than after uncertain cues (i.e., in the uncertain condition), and this effect began from approximately 200 ms after stimulus onset until 750 ms after. Similarly, our previous studies observed more positive responses to certain compared to uncertain negative pictures starting from approximately 200 ms to 1,000 ms (Lin et al., 2015a, 2017b, 2018). When individuals were uncertain about the positive or negative valence of the consequences, our previous study also found that the effect of cue uncertainty occurred as early as 130 ms after pictorial onset (Lin et al., 2015a). Given that more positive-going ERP responses to emotionally (un)certain events are thought to be associated with increased attention to relevant events (Dieterich et al., 2016, 2017), the findings suggest that certain cues enhance attention towards emotional events, particularly negative events, during both perceptual and evaluation processes.

Regarding emotional cues, only one ERP study, to the best of our knowledge, has investigated the effects of cue uncertainty (Yang et al., 2012). In that study, emotional pictures and simple neutral symbols (e.g., “+”) were used as certain and uncertain cues, respectively. The authors showed that compared to uncertain cues, certain cues led to a less positive-going ERP response to both fearful and neutral faces at approximately 150 ms, suggesting that certain cues decrease attention during the perceptual processing of emotional faces. Yang et al. (2012) also reported an interaction between cue uncertainty and facial expression even in very early time ranges (<100 ms), but the authors did not report the specific effects of cue uncertainty in each category of facial expression. The findings in the present study might suggest that certain cues reduce attention towards emotional events only in perceptual processes.

However, Yang et al. (2012) did not investigate the effects of cue uncertainty in later evaluation processes. Due to the lack of analysis of these processes, the study does not fully clarify the influence of cue uncertainty on emotional events when emotional cues are used. More importantly, Yang et al. (2012) used emotional pictures and neutral signs as certain and uncertain cues, respectively. Those two groups of stimuli were different not only in emotional features but also in emotionally irrelevant physical features (e.g., stimulus size and complexity). Accordingly, participants in that study could have used emotional and/or non-emotional physical features of the cues to predict the emotional consequences. It was therefore uncertain whether the effects of cue uncertainty shown in that study were associated with non-emotional or emotional features of the cues (or both classes of features). Thus, the first aim of the present study was to use emotional cues with similar physical features to further investigate the effects of cue uncertainty on ERPs, particularly in later evaluation processes.

Moreover, the effect of cue uncertainty in perceptual stages in Yang et al.’s (2012) study was different from the effect of non-emotional cues (Gole et al., 2012; Lin et al., 2015a, 2017b, 2018). These differential findings might imply that the effects of cue uncertainty, at least these effects in perceptual stages, are different for emotional and non-emotional cues. Nevertheless, considering the limitations of Yang et al.’s (2012) study and some other differences among the studies regarding non-emotional and emotional cues (e.g., pictorial and facial targets), it remains uncertain whether the differential effects of cue uncertainty in emotional and non-emotional cues were confounded by these limitations and/or differences. Thus, the second aim of the present study was to directly investigate whether cue valence (i.e., emotional and non-emotional cues) influences the effects of cue uncertainty on ERP responses to emotional events. Investigation of the influence of cue valence might provide new theoretical insights into expectancy bias, i.e., under what circumstances attention is enhanced or reduced by certain cues.

Thus, participants in the present study were asked to view positive and negative pictures that were preceded by expectancy cues. The cues either indicated the emotional consequence of the pictures (i.e., certain cues) or did not (i.e., uncertain cues). In the emotional cue condition, the cues were emotional faces. The emotionally certain and uncertain consequences were signified by the emotion of the facial expressions. To reduce the differences in physical features between certain and uncertain cues, the present study used facial stimuli for all cues: happy and fearful faces were used as certain cues for upcoming positive and negative pictures, respectively, and neutral faces were used as uncertain cues. In the non-emotional cue condition, the expectancy cues were scrambled faces that were outlined in different colors. Certain and uncertain consequences were indicated by the colors of the outlines: red and blue outlines indicated upcoming positive and negative pictures, respectively, and green outlines indicated that the emotional content of the upcoming pictures was uncertain. Using scrambled faces as cues reduced differential physical features with facial cues.

Notably, previous studies have often included neutral pictures in such experiments (Gole et al., 2012; Dieterich et al., 2016, 2017; Lin et al., 2017b, 2018; Qiao et al., 2018). If we had done the same in the present study, neutral faces should have been used as certain cues in the emotional cue condition. However, these faces also served as uncertain cues in the present study. If neutral expressions were used for both purposes, participants would not know whether the faces indicated neutral or emotionally uncertain pictures to come. With the limited information available, we failed to find a solution to this issue and thus did not include neutral pictures in the present study.

Theories and related empirical studies on expectancy bias have suggested that cues indicating emotional consequences pre-activate the emotion corresponding to the consequence (e.g., Tomarken et al., 1989; de Jong et al., 1995; Davey and Dixon, 1996; Sussman et al., 2017), which influences attention to the upcoming consequence. When the processing of cues (e.g., positive cues) does not require large attentional resources, this pre-activation might help in enhancing vigilance and attention towards emotional events. Otherwise, when the cues (e.g., negative faces) require large attentional resources, the pre-activated attentional resources might have been used for cue processing. Moreover, the processing of these cues might also occupy attentional resources allocated to the events after their occurrences. Due to limited resources, attention allocated to the events might be reduced. Given that increased attention to emotionally (un)certain events are associated with positive-going ERP responses (Dieterich et al., 2016, 2017), our first hypothesis of the present study is that for positive pictures, ERP responses might shift to a more positive direction in the certain condition than in the uncertain condition at perceptual and evaluation processes when the cues are emotional. In contrast, certain cues might result in more negative-going ERP responses to negative pictures than uncertain cues in related processes. Moreover, in light of our first hypothesis and previous studies regarding non-emotional cues (Gole et al., 2012; Yang et al., 2012; Lin et al., 2015a, 2017b, 2018; Dieterich et al., 2016, 2017; Qiao et al., 2018), our second hypothesis is that cue valence would modulate the effects of cue uncertainty on ERP responses to emotional events. Specifically, the effect of cue uncertainty will be reversed between the emotional cue and non-emotional cue conditions, particularly for negative pictures.

## Materials and Methods

### Participants

Twenty-two healthy participants were recruited from the undergraduate and postgraduate student population of universities in Guangzhou city *via* advertisements. Two participants were excluded from the statistical analysis due to excessive artifacts in electroencephalograph (EEG) signals, resulting in a total of 20 participants (18–31 years old, *M* = 21.66, *SD* = 3.22; 11 females). Participants were right-handed, as determined by the Edinburgh Handedness Inventory (Oldfield, 1971). Participants had normal or corrected-to-normal vision, and none of them reported a history of neurological illness. All participants gave informed consent following standard ethical guidelines as defined in the Declaration of Helsinki. The study was approved by the academic committee of the School of Public Administration, Guangdong University of Finance.

### Stimuli

The stimuli in the present study included pictures that were obtained from the International Affective Picture System (IAPS; Lang et al., 2008), pictures of faces, and pictures of scrambled faces. The IAPS pictures consisted of 120 colored pictures (60 positive and 60 negative^1^). No human faces were included in these pictures. All IAPS pictures were adjusted to a size of 9.03 cm × 6.77 cm (horizontal × vertical) and a resolution of 28.35 pixels/cm, and they were aligned in luminance, contrast, and composition. The selection of these pictures was based on normative valence and arousal ratings reported in Lang et al.’s (2008) study. According to the normative valence ratings, positive pictures (*M ± SD* = 6.96 ± 0.66) were rated as more pleasant than negative pictures (3.13 ± 0.72; *F*_(1,118)_ = 929.11, *p* < 0.001, $\eta_{\text{p}}^{2}$ = 0.89). The ratings for negative and positive pictures had similar distances from the neutral value [i.e., 5; (5 − negative ratings) vs. (positive ratings – 5) = 1.96 ± 0.66 vs. 1.87 ± 0.72 ; *F*_(1,118)_ = 0.62, *p* = 0.434, $\eta_{\text{p}}^{2}$ = 0.01]. The arousal ratings were similar between these two categories of pictures (positive vs. negative = 5.13 ± 0.94 vs. 5.17 ± 0.76; *F*_(1,118)_ = 0.09, *p* = 0.763, $\eta_{\text{p}}^{2}$ < 0.01).

For faces, the present study included two sets of 60 colored facial stimuli. The two sets of faces were the same. All of these facial pictures were made by FACSGen 2.0 animation software (Roesch et al., 2006). For each set, the facial pictures portrayed 20 Asian individuals (10 males and 10 females; 20–35 years old) with a fearful, happy and neutral expression each. The facial pictures were cropped similarly around the face outline and centered so that the eyes, noses, and mouths were at similar positions. Shoulders and hair were removed. The facial pictures were adjusted to a size of 5.29 cm × 5.29 cm with a resolution of 28.35 pixels/cm, and they aligned in luminance, contrast, hue, and color. The background color was set to black. For one set of faces, the outlines were depicted in white. For the other set, the outlines of happy, fearful and neutral faces were depicted in red, blue and green, respectively. The faces excluding the outlines were then scrambled using the Scrambled Tool in Adobe Photoshop CS6.

The faces (rather than scrambled faces) were rated on valence (ranging from 1 to 9, 1 = very unpleasant, 9 = very pleasant) and arousal (ranging from 1 to 9, 1 = very low, 9 = very high) by an independent sample of 32 participants (20 females; 19–26 years, *M* = 21.09, *SD* = 2.10) in our prior study. We performed a repeated-measures ANOVA with the factor of facial expression (fearful, happy and neutral) separately for valence and arousal ratings. The results with regard to valence ratings revealed a main effect of facial expression (*F*_(2,62)_ = 161.49, Greenhouse-Geisser epsilon = 0.60, corrected *p* < 0.001, $\eta_{\text{p}}^{2}$ = 0.84). *Post hoc* *t*-tests showed that happy faces (6.07 ± 0.76) were rated as more pleasant than neutral (4.75 ± 0.31) and fearful faces (3.35 ± 0.64), and neutral faces were rated as more pleasant than fearful faces (*p* < 0.001; all *p*-values were corrected by Bonferroni correction). For arousal ratings, the effect of facial expression was also significant (*F*_(2,62)_ = 35.60, Greenhouse-Geisser epsilon = 0.79, corrected* p* < 0.001, $\eta_{\text{p}}^{2}$ = 0.54). *Post hoc* *t*-tests showed that the arousal ratings were higher for fearful (5.74 ± 0.16) and happy faces (5.57 ± 0.18) than for neutral faces (4.00 ± 0.22, *p* < 0.001), and there were no differences between fearful and happy faces (*p* > 0.05; all *p*-values were corrected by Bonferroni correction).

### Procedure

After participants’ informed consent was obtained and their handedness was determined, they were asked to sit in a soundproof and dimly lit room. The participants were told that they would be presented with an emotional or a scrambled face and followed by a picture and that they would then rate the picture on a 9-point scale ranging from “1” (extremely unpleasant) to “9” (extremely pleasant) without a time limit on their response. Participants were told that faces were always outlined in white, while scrambled faces were outlined in red, blue or green. They were informed that fearful faces and scrambled faces with blue outlines indicated an upcoming negative picture, happy faces and scrambled faces with red outlines indicated an upcoming positive picture, and neutral faces and scrambled faces with green outlines indicated that the emotional content of the upcoming picture was uncertain.

**Figure 1:**
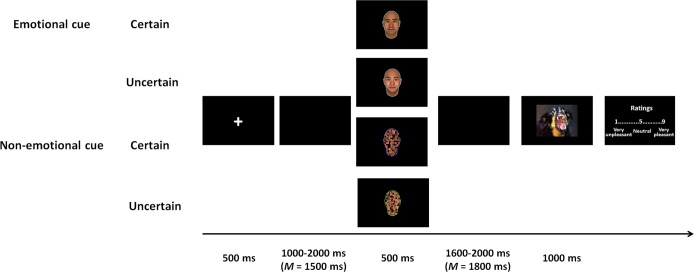
Experimental procedure. Negative pictures were presented after certain or uncertain cues in either the emotional cue condition or the non-emotional cue condition.

Stimuli were presented using E-Prime 1.0 software (Psychology Software Tools, Inc., Sharpsburg, PA, USA) on a black screen in the center of a 17″ monitor with a screen resolution of 1,024 × 768 pixels. The viewing distance was approximately 80 cm, so the visual angles of the IAPS and facial/scrambled pictures were 6.46° × 4.85° and 3.79° × 3.79°, respectively. All stimuli were shown against a dark background. As illustrated in Figure 1, each trial began with a fixation cross for 500 ms, followed by a blank screen varying from 1,000 ms to 2,000 ms (*M* = 1,500 ms). Subsequently, cues were presented at the center of the screen for 500 ms. In the emotional cue condition, fearful and happy faces served as cues signifying an upcoming negative and positive picture, respectively, and neutral faces served as uncertain cues. In the non-emotional cue condition, certain positive and negative cues were represented by scrambled faces with blue and red outlines, respectively, and uncertain cues were represented by scrambled faces with green outlines. For both the emotional and non-emotional cue conditions, uncertain cues were followed by a positive or a negative picture with equal conditional probabilities. After a blank screen was shown for 1,600 ms to 2,000 ms (*M* = 1,800 ms), a positive or a negative picture was shown for 1,000 ms. After another blank screen was shown for 500 ms, a 9-point scale was presented to instruct participants to rate the pictures (ranging from 1 to 9, “1” = very unpleasant, “5” = neutral, “9” = very pleasant). The next trial began immediately after the ratings were given.

After the task, the participants were asked to estimate the frequencies of negative pictures in the uncertain condition, as previous studies showed that individuals overestimated the occurrences of uncertain negative pictures (Sarinopoulos et al., 2010; Grupe and Nitschke, 2011; Dieterich et al., 2016; Lin et al., 2018). According to cue valence (an emotional cue vs. a non-emotional cue), cue uncertainty (certain vs. uncertain) and emotion (positive vs. negative), the present study contained eight experimental conditions (i.e., emotional cue—certain—positive, emotional cue—certain—negative, emotional cue—uncertain—positive, emotional cue—uncertain—negative, non-emotional cue—certain—positive, non-emotional cue—certain—negative, non-emotional cue—uncertain—positive and non-emotional cue—uncertain—negative). All experimental conditions were presented in a randomized order. Each of the 60 positive and 60 negative pictures listed in Footnote 1 of the *Stimuli* section was presented four times, with the emotional cue—certain condition, the emotional cue—uncertain condition, the non-emotional cue—certain condition and the non-emotional cue—uncertain condition for each. Therefore, there were 60 trials for each experimental condition, resulting in a total of 480 trials. Before the actual experiment, participants had to perform 24 practice trials to familiarize themselves with the procedure. The complete task lasted for approximately 1 h.

### Behavioral Recordings

For the behavioral data, the pleasantness ratings of the pictures were recorded. Post-experiment frequency estimation of uncertain negative pictures was also recorded.

### EEG Recordings

EEG was continuously recorded using a Neuroscan Synamps2 AC-amplifier (NeuroScan Inc., Sterling, VA, USA). Ag/AgCl electrodes were placed on the scalp by using a 32-channel cap following the international extended 10-20 system. EEG electrodes were connected to the ground and referenced to the right mastoid online. The horizontal electrooculogram (EOG) was monitored from two electrodes at the outer canthi of both eyes, and the vertical EOG was recorded bipolarly from electrodes above and below the right eye. The EEG was amplified using a 0.05–100 Hz bandpass filter and a 50 Hz notch filter and sampled at 500 Hz/channel. Electrode impedances were maintained below 5 kΩ.

Offline, the EEG data were analyzed using SCAN 4.5 software. Raw EEG signals were digitally re-referenced to the average of both the left and right mastoids. Ocular movements were inspected and removed using a regression procedure implemented with NeuroScan 4.5 (Semlitsch et al., 1986). The EEG data were then segmented into 1,200 ms from −200 to 1,000 ms relative to picture onset, with the first 200 ms serving as a baseline. Artifact rejection was implemented with an amplitude threshold of 100 μV. Trials were averaged separately for each channel and experimental condition and averaged ERPs were digitally low-pass filtered at 30 Hz (24 db/oct, zero phase shift, Butterworth).

ERPs for the pictures were quantified using ERP amplitudes in the time window of 60–1,000 ms. The amplitudes were measured at the frontal (F3, Fz, F4), frontal-central (FC3, FCz, FC4), central (C3, Cz, C4), central-parietal (CP3, CPz, CP4) and parietal (P3, Pz, P4) electrodes. Time windows and electrodes of interest were chosen based on a visual inspection of the grand-mean waveforms and previous studies (e.g., Gole et al., 2012; Lin et al., 2012, 2014b, 2015a, 2017b; Yang et al., 2012).

### Data Analysis

**Figure 2:**
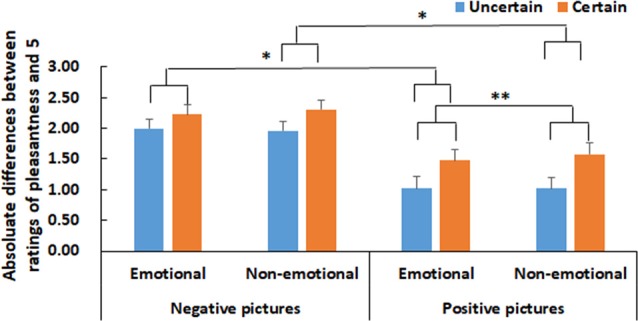
Absolute differences between pleasantness ratings and the neutral value (i.e., 5) for pictures in each experimental condition. Vertical lines indicate the *SE* of the mean. The significance level of the effect of cue uncertainty is marked by the number of “*” symbols. “*” and “**” indicate *p* < 0.05 and 0.01, respectively.

For behavioral data, the estimated frequency of uncertain negative pictures was analyzed by a one-sample *t*-test with a test value of 0.50 for each level of cue valence separately. The estimated frequency was then analyzed with a repeated-measures ANOVA with cue valence (emotional vs. non-emotional) as a within-subject factor. The absolute differences between the value of pleasantness ratings and the neutral value (i.e., 5) were calculated (i.e., negative pictures: 5 minus the value of ratings; positive pictures: the value of ratings minus 5). These differences were analyzed using a 2 × 2 × 2 repeated-measures ANOVA with cue valence (emotional vs. non-emotional), cue uncertainty (uncertain vs. certain) and emotion (positive vs. negative) as within-subject factors. The *mean* and *SE* of the absolute differences for each condition are presented in Figure 2.

About ERPs, we averaged the amplitudes separately for frontocentral (i.e., F3, Fz, F4, FC3, FCz, FC4, C3, Cz, C4) and centroparietal (i.e., CP3, CPz, CP4, P3, Pz, P4) electrodes, as the effect of cue uncertainty was found to differ between frontocentral and centroparietal scalp sites (Lin et al., 2018). For frontocentral and centroparietal ERPs, the mean amplitudes were assessed separately using the abovementioned 2 × 2 × 2 ANOVA, similar to the ratings of pleasantness. A probability level of *p* < 0.05 was considered statistically significant. Only significant effects are reported herein.

## Results

### Behavioral Results

#### Frequency Estimation of Uncertain Negative Pictures

The estimated frequency of uncertain negative pictures was significantly larger than 0.50 (emotional cue: *M* = 0.57, *SD* = 0.12, *t*_(19)_ = 2.57, *p* = 0.019, *d’* = 0.57; non-emotional cue: *M* = 0.59, *SD* = 0.09, *t*_(19)_ = 4.49, *p* < 0.001, *d’* = 0.97). In the ANOVA, the effect of cue valence was not significant (*p* > 0.05).

#### Absolute Differences Between Pleasantness Ratings and the Neutral Value

Pleasantness ratings, in which higher scores mean more extreme ratings with positive pictures but less extreme ratings with negative pictures, might be difficult to understand. To understand the effects regarding pleasantness ratings more clearly, we used the absolute differences between the values of pleasantness ratings and of the neutral rating (i.e., 5), in which higher scores mean more extreme ratings for both negative and positive pictures, to investigate the related issue.

As shown in Figure 2, there were significant main effects of cue uncertainty (*F*_(1,19)_ = 6.45, *p* = 0.020, $\eta_{\text{p}}^{2}$ = 0.25) and emotion (*F*_(1,19)_ = 18.34, *p* < 0.001, $\eta_{\text{p}}^{2}$ = 0.49). The ratings were higher for certain compared to uncertain pictures and for negative compared to positive pictures.

The interaction between cue uncertainty and emotion was significant (*F*_(1,19)_ = 11.62, *p* = 0.003, $\eta_{\text{p}}^{2}$ = 0.38). Further analysis showed that for negative pictures, there was no significant effect of cue uncertainty (*p* > 0.05), whereas for positive pictures, the ratings were higher in the certain condition than in the uncertain condition (*F*_(1,19)_ = 8.84, *p* = 0.008, $\eta_{\text{p}}^{2}$ = 0.32). Another direction of further analysis to resolve the two-way interaction showed that the ratings were higher for negative pictures than for positive pictures in both the certain and uncertain conditions, although the emotional effect was smaller in the certain condition than in the uncertain condition (certain: *F*_(1,19)_ = 14.27, *p* = 0.001, $\eta_{\text{p}}^{2}$ = 0.43; uncertain: (*F*_(1,19)_ = 21.63, *p* < 0.001, $\eta_{\text{p}}^{2}$ = 0.53).

There was also an interaction between cue valence and uncertainty (*F*_(1,19)_ = 5.45, *p* = 0.031, $\eta_{\text{p}}^{2}$ = 0.22). Further analysis showed that the ratings were higher for emotionally certain compared to uncertain pictures in both the emotional and non-emotional cue conditions, although the effect of cue uncertainty was smaller in the emotional cue condition than in the non-emotional cue condition (emotional: *F*_(1,19)_ = 5.60, *p* = 0.029, $\eta_{\text{p}}^{2}$ = 0.23; non-emotional: *F*_(1,19)_ = 7.03, *p* = 0.016, $\eta_{\text{p}}^{2}$ = 0.27). Another direction of further analysis to resolve the two-way interaction showed that in the certain condition, the ratings were higher when the pictures were preceded by non-emotional cues than when they were preceded by emotional cues (*F*_(1,19)_ = 6.75, *p* = 0.018, $\eta_{\text{p}}^{2}$ = 0.26), but the effect of cue valence was not significant in the uncertain condition (*p* > 0.05).

### ERP Results

**Figure 3:**
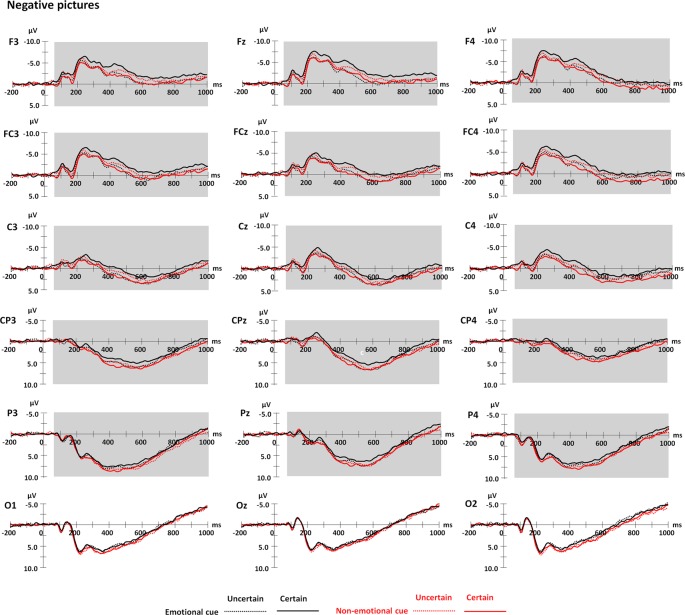
Grand average waveforms for negative pictures varying in cue valence, cue uncertainty, and emotion. Shaded areas represent the time window for frontocentral and centroparietal ERPs (60–1,000 ms).

**Figure 4:**
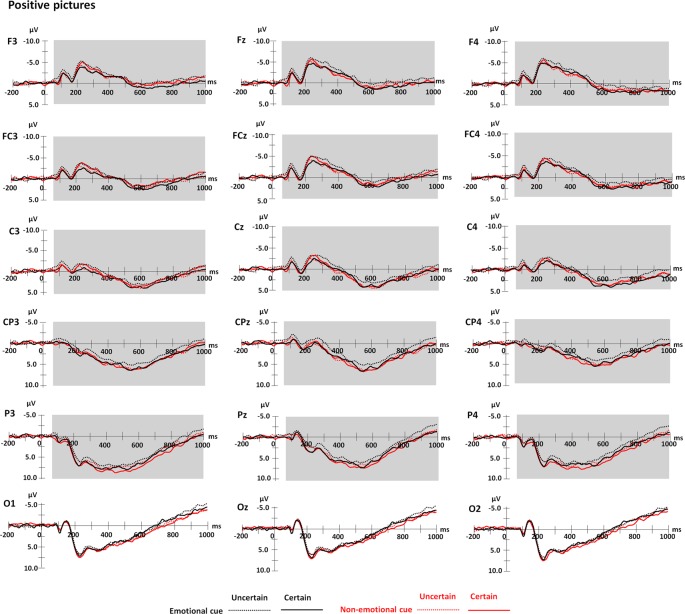
Grand average waveforms for positive pictures varying in cue valence, cue uncertainty, and emotion. Shaded areas represent the time windows for frontocentral and centroparietal ERPs (60–1,000 ms).

**Figure 5:**
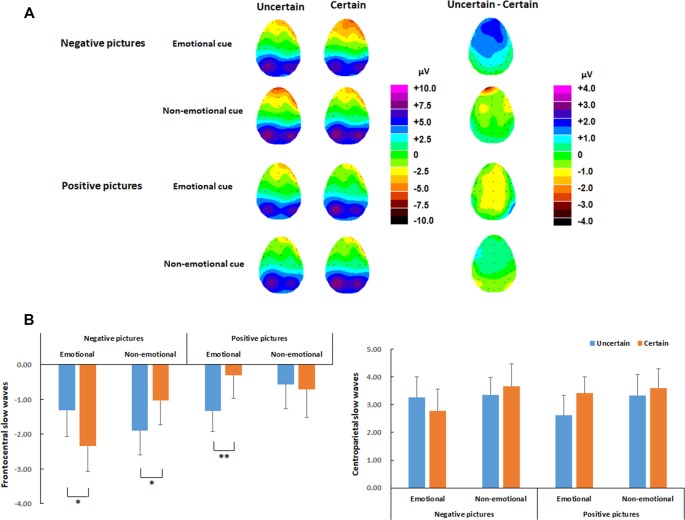
**(A)** Topographical maps based on the mean amplitudes of frontocentral and centroparietal ERPs (60–1,000 ms) for the pictures for each experimental condition and the differences between the uncertain and certain conditions given each cue valence and emotion. **(B)** The means and *SE* values of amplitudes regarding frontocentral and centroparietal ERPs for the pictures in each experimental condition. See the meaning of “*” in the caption of Figure 2.

Grand means of ERP waveforms are presented in Figure 3 for negative pictures and Figure 4 for positive pictures, and the related topography maps and means and *SE* of the amplitudes are presented in Figure 5. Figure 3 suggests that for negative pictures, ERP responses in the time range from 60 to 1,000 ms were shifted to a more negative direction in the emotional cue—certain conditions than in the other conditions. Figure 4 suggests that for positive pictures, the response in the related time range was more negative-going in the emotional cue—uncertain condition than in the other conditions. Moreover, as shown by these figures, the effects for both negative and positive pictures seemed to be stronger over frontocentral scalp sites than centroparietal scalp sites. In the following statistical analyses, we will focus particularly on the relevant effects of cue uncertainty over frontocentral scalp sites.

#### Frontocentral ERPs

There was a main effect of emotion (*F*_(1,19)_ = 21.93, *p* < 0.001, $\eta_{\text{p}}^{2}$ = 0.54). The amplitude was generally more positive for positive pictures than for negative pictures.

More importantly, the three-way interaction among cue valence, cue uncertainty and emotion was significant (*F*_(1,19)_ = 23.78, *p* < 0.001, $\eta_{\text{p}}^{2}$ = 0.56). To understand the effect of cue uncertainty on the influence of the other two factors, separate analysis for each emotional category showed an interaction between cue valence and uncertainty for both negative and positive pictures (negative: *F*_(1,19)_ = 18.33, *p* < 0.001, $\eta_{\text{p}}^{2}$ = 0.49; positive: *F*_(1,19)_ = 11.94, *p* = 0.003, $\eta_{\text{p}}^{2}$ = 0.39). Further analysis showed that for negative pictures, the amplitude was more negative-going in the certain condition than in the uncertain condition when the cues were emotional (*F*_(1,19)_ = 5.60, *p* = 0.029, $\eta_{\text{p}}^{2}$ = 0.23), whereas the amplitude was less negative-going in the certain condition than in the uncertain condition when the cues were non-emotional (*F*_(1,19)_ = 6.14, *p* = 0.023, $\eta_{\text{p}}^{2}$ = 0.24). For positive pictures, the amplitude was more negative-going in the uncertain condition than in the certain condition when the cues were emotional (*F*_(1,19)_ = 9.26, *p* = 0.007, $\eta_{\text{p}}^{2}$ = 0.33), but the effect of cue uncertainty was not significant in the non-emotional cue condition (*p* > 0.05). The other approach to understand the effect of cue uncertainty by using a separate analysis for each level of cue valence showed that there was an interaction between cue uncertainty and emotion in the emotional cue condition (*F*_(1,19)_ = 11.51, *p* = 0.003, $\eta_{\text{p}}^{2}$ = 0.38). In the emotional cue condition, the interaction occurred because the effect of cue uncertainty for negative pictures was reversed with the effect for positive pictures (see the statistical values above). In contrast, in the non-emotional cue condition, the interaction between cue uncertainty and emotion was not significant (*p* > 0.05).

Regarding the emotional effect on the influence of the other two factors, separate analysis for each level of cue uncertainty showed an interaction between cue valence and emotion in both the certain and uncertain conditions (certain: *F*_(1, 19)_ = 12.64, *p* = 0.002, $\eta_{\text{p}}^{2}$ = 0.40; uncertain: *F*_(1,19)_ = 7.97, *p* = 0.011, $\eta_{\text{p}}^{2}$ = 0.30). In a certain condition, the amplitude was more negative-going for negative pictures than for positive pictures when the cues were emotional (*F*_(1,19)_ = 29.77, *p* < 0.001, $\eta_{\text{p}}^{2}$ = 0.61), whereas the emotional effect was not significant when the cues were non-emotional (*p* > 0.05). For the uncertain condition, there was no emotional effect when the cues were emotional (*p* > 0.05), whereas the amplitude was more negative-going for negative pictures than for positive pictures when the cues were non-emotional (*F*_(1,19)_ = 12.10, *p* = 0.003, $\eta_{\text{p}}^{2}$ = 0.39). The other approach to understanding the emotional effect using a separate analysis for each level of cue valence showed that there was an interaction between cue uncertainty and emotion in the emotional cue condition(see the statistical values above). This significant interaction was due to a significant emotional effect in a certain condition and an insignificant effect in the uncertain condition (see the statistical values above). For the non-emotional cue condition, the interaction between cue uncertainty and emotion was not significant (see the statistical values above).

For the effect of cue valence on the influence of the other two factors, separate analysis for each level of cue uncertainty showed an interaction between cue valence and emotion in both the certain and uncertain conditions. Further analysis showed that in the certain condition, the amplitude elicited by negative pictures was more negative in the emotional cue condition than in the non-emotional cue condition (*F*_(1,19)_ = 9.43, *p* = 0.006, $\eta_{\text{p}}^{2}$ = 0.33), whereas the effect of cue valence was not significant for positive pictures (*p* > 0.05). In the uncertain condition, the effect of cue valence was not significant for negative pictures (*p* > 0.05), but for positive pictures, the amplitudes were more negative in the emotional cue condition than in the non-emotional cue condition (*F*_(1,19)_ = 5.44, *p* = 0.031, $\eta_{\text{p}}^{2}$ = 0.22). The other approach to understanding the effect of cue valence using a separate analysis for each emotional category showed an interaction between cue valence and uncertainty for both negative and positive pictures (see the statistical values above). As suggested above, the interaction for negative pictures was due to a significant effect of cue valence in the certain condition and an insignificant effect in the uncertain condition (see the statistical values above). For positive pictures, the interaction was due to an insignificant effect in certain conditions and a significant effect in the uncertain condition (see the statistical values above).

#### Centroparietal ERPs

There were no significant main effects or interactions (*p* > 0.05).

## Discussion

In the present study, we aimed to gain a better understanding of the effects of cue uncertainty on ERP responses to emotional events when the emotional consequences were indicated by emotional cues; measures were taken to reduce the differences in physical features between certain and uncertain cues. Also, we investigated whether cue valence influenced the effects of cue uncertainty on ERP responses to emotional events. The results showed that for the emotional cue condition, ERP responses elicited by negative pictures were more negative in certain conditions than in the uncertain condition starting from 60 ms to the offset of the pictures. For positive pictures, ERP responses were less negative in the certain condition than in the uncertain condition. The findings may indicate that certain cues alter attention in perceptual and evaluation stages of emotional events when the events are signified by emotional cues. More importantly, the findings also show that the effects of cue uncertainty differ between non-emotional cue and emotional cue conditions in relevant processes, possibly indicating that cue valence modulates the effects of cue uncertainty on attention to emotional events.

### The ERP Effects of Cue Uncertainty in the Emotional Cue Condition

The findings in the emotional cue condition were in line with our hypotheses. For negative pictures, the findings might suggest that certain compared to uncertain cues reduce attention allocated to relevant pictures during perceptual and evaluation stages. Regarding positive pictures, the findings might indicate that certain cues enhance attention to the pictures in relevant stages.

When the cues indicate upcoming emotional consequences, the cues might pre-activate the attentional mechanisms before the consequences and enhance vigilance attention afterward (e.g., Tomarken et al., 1989; de Jong et al., 1995; Davey and Dixon, 1996; Lin et al., 2012, 2014b; Aue et al., 2013; Sussman et al., 2017). While uncertain cues also enhance attention to upcoming events (e.g., Grupe and Nitschke, 2011, 2013; Dieterich et al., 2016, 2017; for a review, Anselme, 2010), the enhanced attention elicited by certain cues is thought to be stronger than that elicited by uncertain cues in the context of implicit expectations (Lin et al., 2018). Thus, in the present study, positive facial cues occurring in the context of implicit expectations might pre-activate the participants’ attentional mechanisms during the presentations and subsequently enhance their attention to certain positive events.

Similar to that for positive pictures, the pre-activation of attention mechanisms might also occur in the negative condition. However, in contrast to certain positive cues (i.e., happy faces), certain negative cues (i.e., fearful faces) might capture more attention due to negativity bias (Carretié et al., 2001; Huang and Luo, 2006). The processing of these certain negative cues might have used attentional resources associated with pre-activation. Moreover, the processing might even have occupied attentional resources allocated to certain negative pictures (Flaisch et al., 2008; Lin et al., 2015c, 2017a). Because there are limited resources, attentional resources allocated towards relevant pictures might be reduced. An alternative explanation might be that different from other cues (non-emotional—negative/positive cues and emotional—positive cues), fearful facial cues also pre-activate specific executive functions to handle the challenges (e.g., subsequent negative pictures) to the organism (Pessoa, 2009). This pre-activation might reduce the impact of negative pictures and thus attention towards negative pictures. Consistent with this explanation, previous studies showed that executive control reduced emotional responses to negative events (e.g., Cohen et al., 2015a,b, 2016).

Moreover, the findings of the present study were partially in line with the findings of previous studies associated with emotional priming. For example, preceding negative stimuli showed more negative-going ERP responses to target stimuli at perceptual and evaluation stages (e.g., Furl et al., 2007; Flaisch et al., 2008; Hietanen and Astikainen, 2013; Wieser et al., 2014; Lin et al., 2015b,c). For positive pictures, positive priming elicited positive-going responses at perceptual stages (e.g., Flaisch et al., 2008; Hietanen and Astikainen, 2013). However, studies regarding emotional priming did not report an effect at perceptual stages in which the time range was shorter than 100 ms (Furl et al., 2007; Flaisch et al., 2008; Hietanen and Astikainen, 2013; Wieser et al., 2014; Lin et al., 2015c). Also, ERP responses were more negative-going for positive target stimuli preceded by positive primes in evaluation processes (Hietanen and Astikainen, 2013). These findings suggest that the processing mechanisms underlying the effects of cue uncertainty in the emotional cue condition are not the same as the mechanisms underlying the effects of emotional priming.

Additionally, the findings in the present study were consistent with the findings of Yang et al. (2012), who observed more negative-going ERP responses to fearful faces preceded by certain than uncertain cues in perceptual processes. However, Yang et al. (2012) did not report an effect of cue uncertainty in evaluation processes. As mentioned in the “Introduction” section, the study by Yang et al. (2012) investigated the effects of cue uncertainty by using certain and uncertain cues (e.g., an emotional picture and a neutral symbol “+,” respectively) that differed in both emotional features and non-emotional physical features. In the present study, certain and uncertain cues differed only in emotional as opposed to non-emotional features. The results showed that cue uncertainty influenced neural responses to emotional events even in evaluation processes when the consequence is signified only by the emotional features of the cues.

### Influence of Cue Valence on the ERP Effects of Cue Uncertainty for Emotional Events

The findings regarding the modulatory effects of cue valence were in line with the hypotheses, which suggested that cue valence will influence the effects of cue uncertainty on ERP responses in perceptual and evaluation processes. Specifically, for negative pictures, the effects on ERP responses in the emotional cue condition (see the specific effects in “The ERP Effects of Cue Uncertainty in the Emotional Cue Condition” section) significantly differed from those in the non-emotional cue condition, i.e., there were fewer negative-going responses to certain negative pictures than uncertain negative pictures in the perceptual and evaluation processes. Regarding positive pictures, there were effects of cue uncertainty in the emotional cue condition (see the specific effects in “The ERP Effects of Cue Uncertainty in the Emotional Cue Condition” section), whereas the effects were not significant for the non-emotional cue condition. The findings may suggest that cue valence modulates the effects of cue uncertainty on emotional events relating to attention allocation, with the nature of the modulation depending on the valence of the events.

For negative pictures, the findings in the present study may suggest that in the emotional cue condition, attention is shifted away from certain negative pictures in the perception and evaluation stages. However, in the non-emotional cue condition, attention might be shifted towards certain negative pictures. The differential effects of cue uncertainty between emotional and non-emotional cue conditions might be due to two different processing mechanisms in the certain condition. As mentioned in sections “The ERP Effects of Cue Uncertainty in the Emotional Cue Condition”, in the emotional cue condition, the processing of certain cues (i.e., fearful faces) might have used the pre-activated attentional resources. Moreover, the processing might also occupy large attentional resources allocated to negative pictures, resulting in reduced attention to the pictures. In the non-emotional cue condition, however, certain cues used by scrambled faces are less salient and thus do not require large attentional resources. These cues might be beneficial for enhancing vigilance attention to subsequent emotional events, similar to positive facial cues. An alternative explanation is that fearful facial cues in the emotional cue condition might enhance executive control (e.g., Cohen et al., 2015a,b, 2016), whereas executive control might not be enhanced by scrambled facial cues. The differential degree of executive control between the emotional and non-emotional cue conditions might lead to differential impacts on attention to negative events.

For positive pictures, there was a modulation of cue valence on cue uncertainty, as there were effects of cue uncertainty in the emotional cue condition, but the effects were not significant in the non-emotional cue condition. As mentioned in sections “The ERP Effects of Cue Uncertainty in the Emotional Cue Condition,” the effect of cue uncertainty in the emotional cue condition might be associated with pre-activated attentional elicited by certain cues (i.e., positive faces). For the non-emotional cue condition, the ERP differences between certain and uncertain positive pictures might be associated with enhanced attention allocated to uncertain positive pictures. It has been shown that after receiving uncertain cues, individuals might be biased toward expecting the occurrence of negative events (Grupe and Nitschke, 2011, 2013; Dieterich et al., 2016, 2017; Lin et al., 2018). According to section “Frequency Estimation of Uncertain Negative Pictures” in the present study, this bias also occurred in the present study. This bias might produce slight expectation violations to some extent after the occurrence of positive events. Solving this expectation violation might require the engagement of attention. Moreover, as mentioned in the “Introduction” section, uncertain cues could recruit attentional resources to process upcoming events, but this degree is not strong in the context of implicit expectations (Grupe and Nitschke, 2011, 2013; Dieterich et al., 2016, 2017). However, compared to uncertain cues in the emotional cue condition (neutral faces), those cues in the non-emotional cue condition (i.e., scrambled faces) are less salient. The degree of activating attention mechanisms after uncertain cues might be weaker in the non-emotional cue condition and thus might be insufficient to address even slight expectation violations. In this case, participants might have to recruit additional attentional resources to solve this expectation violation.

The results also showed that cue valence did not modulate the effect of cue uncertainty on ERP amplitude over centroparietal scalp sites irrespective of pictured emotion, as the effects of cue uncertainty were not significant for either the emotional or non-emotional cue condition. Our previous study showed that the effect of cue uncertainty on centroparietal ERP response was evident only in the context of explicit expectations (Lin et al., 2018). As the present study was investigated in the context of implicit expectations, the lack of a significant effect of cue uncertainty on centroparietal ERP responses was not surprising.

It seemed that the effect of cue uncertainty for negative pictures generally occurred earlier than that reported in previous studies (e.g., Gole et al., 2012; Lin et al., 2012, 2014b, 2015a,d, 2017b, 2018; Yang et al., 2012; Dieterich et al., 2016, 2017). These previous studies showed that the effect emerged after 100 ms with stimulus onset, but in the present study, the effect of cue uncertainty started before 100 ms. Participants in previous studies performed the cue-event paradigm in either the non-emotional cue condition or the emotional cue condition. However, in the present study, the cue-event paradigms in both of these conditions were shown randomly to the participants. As additional categories of expectancy cues were presented, participants might have developed an increasingly elaborate process for identifying the cues and recalling their meanings, irrespective of cue valence and uncertainty. The elaborated processing of the cues might cause the effect of cue uncertainty to emerge in earlier time ranges in general. Future studies might present emotional and non-emotional cues in different blocks to further investigate whether the modulation of cue valence begins at such an early time range.

Besides, for the emotional cue—certain condition and non-emotional cue—uncertain conditions, positive compared to negative pictures elicited more positive-going ERP responses. These findings were consistent with previous studies, which have repeatedly shown that ERP responses were shifted to a more positive direction for positive pictures than for negative pictures, possibly suggesting that attention is shifted to positive stimuli (e.g., Smith, 2012 Olofsson et al., 2008; Briggs and Martin, 2009; Dennis, 2010; Leite et al., 2012; Lin et al., 2012, 2015a). As mentioned above, these emotional effects might be associated with reduced attention to negative pictures in the emotional cue—certain condition and enhanced attention to positive pictures in the non-emotional cue—uncertain condition.

### Effects Cue Valence, Cue Uncertainty and Emotion on (Un)pleasantness Ratings

Concerning behavioral data, the findings generally showed that the ratings were more extreme in certain conditions than in the uncertain condition. The findings were in line with those of our previous studies (Lin et al., 2012, 2014b, 2015a, 2017b). The extreme ratings might be because participants in certain conditions tended to rate on the pictures under the guidance of the cues, whereas this was not the case for those participants in uncertain conditions.

Behavioral results also showed that the ratings were more extreme for negative pictures than for positive pictures, irrespective of cue uncertainty and cue valence. For the normative ratings, however, the extreme extents were similar between positive and negative pictures. This issue also seems to occur in several previous studies regarding cue uncertainty (e.g., Lin et al., 2012, 2014b, 2015a, 2017b). A possible explanation for it is that expectancy cues generally alter the (un)pleasantness ratings of the pictures, particularly the negative pictures, irrespective of cue uncertainty and valence. However, due to limited evidence, the mechanisms underlying this issue remain to be investigated in future studies.

### Limitations and Future Directions

Finally, we note several limitations of the present study and suggest outlines for future research. First, in the emotional cue condition, the effect of cue uncertainty might be confounded by the effect of emotional congruency between cue and target emotions, as the cue and target emotions were always congruent in the certain condition and incongruent in the uncertain condition. However, it does not appear possible to exclude this confounding factor in a single experiment. Also, we did not investigate the effects of cue valence and uncertainty for neutral stimuli, as neutral faces could not be used as both certain and uncertain cues (see further details in the “Introduction” section). Future studies might look for ways to address these problems and further investigate related issues. Second, for the interaction among cue valence, cue uncertainty and emotion on ERP responses, the sample size of the present study (*N* = 20) might be too small to achieve adequate power, if the effect size was not large. For example, detecting a medium effect size of $\eta_{\text{p}}^{2}$ in SPSS (i.e., 0.06) would require 35 participants to achieve 0.80 power [conducted by G*Power 3.1.9.2 software, Faul et al., 2007; Statistical test: analysis of variance (ANOVA): repeated measures, within factors; *α* = 0.05; *N* of groups = 1, *N* of measurements = 8, nonsphericity correction = 1; effect size specification: in SPSS]. However, the actual results in the present study revealed an unexpectedly large effect size (i.e., 0.56) for the three-way interaction. In this case, the achieved power was adequate (xxxx 1.00), even though the sample size was small. Nevertheless, future studies might expand the sample size to investigate the related issue. Third, the present study used positive and negative pictures that elicit comparable levels of arousal. Therefore, the present study essentially suggests that the influence of cue valence on cue uncertainty effects is dependent on valence. Future studies may investigate whether this influence is affected by emotional arousal and/or the interaction between valence and arousal. Finally, it has been shown that the effects of cue uncertainty are modulated by several factors, such as the intolerance of cue uncertainty (Gole et al., 2012), attention (e.g., Ran et al., 2016) and obsessive-compulsive symptoms (e.g., Dieterich et al., 2017). As we did not collect data regarding these issues, it is still unknown whether these factors affected the results of the present study. Future studies may collect data regarding related factors and include them as co-variables to further investigate the effects of cue valence.

## Conclusion

In conclusion, the present study might suggest that when an emotional consequence is indicated by the emotional features of cues, certain as compared to uncertain cues reduce attention to negative events and enhance attention to positive events in both perception and evaluation processes. More importantly, the present study might indicate for the first time that cue valence modulates the effects of cue uncertainty on attention to emotional events in relevant processes and that these modulations are dependent on the valence of the events. The present study may help clarify how different categories of expectancy cues affect the processing of emotional events and how such cues help individuals use different expectancy strategies to regulate their responses to subsequent emotional events.

## Data Availability Statement

All datasets generated for this study are included in the article.

## Ethics Statement

The studies involving human participants were reviewed and approved by academic committee of School of Public Administration, Guangdong University of Finance. The patients/participants provided their written informed consent to participate in this study.

## Author Contributions

HL was involved in study design, execution, data analysis, and manuscript drafting and revision. JL, TL, ZL, and HJ were involved in study design, data analysis and manuscript revision. We have read and approved the manuscript and agree to be accountable for all aspects of the work in ensuring that questions related to the accuracy or integrity of any part of the work are appropriately investigated and resolved.

## Conflict of Interest

The authors declare that the research was conducted in the absence of any commercial or financial relationships that could be construed as a potential conflict of interest.

## Footnotes

^1^The numbers of IAPS pictures used in the present study were 1026, 1030, 1033, 1051, 1090, 1111, 1113, 1202, 1274, 1280, 1304, 1410, 1440, 1460, 1463, 1500, 1510, 1560, 1603, 1630, 1650, 1710, 1720, 1731, 1740, 1810, 1811, 1920, 1942, 1947, 2692, 3001, 3216, 5199, 5215, 5300, 5301, 5480, 5594, 5600, 5623, 5626, 5629, 5661, 5825, 5829, 5890, 5900, 5950, 5970, 5972, 5973, 7013, 7023, 7054, 7078, 7079, 7135, 7230, 7238, 7250, 7285, 7330, 7340, 7360, 7380, 7405, 7472, 7477, 7480, 7482, 7521, 7570, 7600, 7650, 8030, 8080, 8179, 8185, 8211, 8400, 8500, 8501, 8502, 8510, 8531, 9000, 9001, 9008, 9010, 9080, 9140, 9181, 9185, 9280, 9290, 9295, 9320, 9340, 9390, 9420, 9426, 9469, 9470, 9471, 9472, 9560, 9561, 9570, 9571, 9610, 9621, 9832, 9900, 9901, 9908, 9911, 9920, 9922, 9941.
